# Foot-and-mouth disease virus-like particles produced by a SUMO fusion protein system in *Escherichia coli* induce potent protective immune responses in guinea pigs, swine and cattle

**DOI:** 10.1186/1297-9716-44-48

**Published:** 2013-07-04

**Authors:** Hui-Chen Guo, Shi-Qi Sun, Ye Jin, Shun-Li Yang, Yan-Quan Wei, De-Hui Sun, Shuang-Hui Yin, Jun-Wu Ma, Zai-Xin Liu, Jian-Hong Guo, Jian-Xun Luo, Hong Yin, Xiang-Tao Liu, Ding Xiang Liu

**Affiliations:** 1 State Key Laboratory of Veterinary Etiological Biology, National Foot and Mouth Disease Reference Laboratory, Lanzhou Veterinary Research Institute, Chinese Academy of Agricultural Sciences, Xujiaping 1, Lanzhou, Gansu 730046, China; 2 School of Biological Sciences, Nanyang Technological University, 60 Nanyang Drive, Nanyang 637551, Singapore

## Abstract

Foot-and-mouth disease virus (FMDV) causes a highly contagious infection in cloven-hoofed animals. The format of FMD virus-like particles (VLP) as a non-replicating particulate vaccine candidate is a promising alternative to conventional inactivated FMDV vaccines. In this study, we explored a prokaryotic system to express and assemble the FMD VLP and validated the potential of VLP as an FMDV vaccine candidate. VLP composed entirely of FMDV (Asia1/Jiangsu/China/2005) capsid proteins (VP0, VP1 and VP3) were simultaneously produced as SUMO fusion proteins by an improved SUMO fusion protein system in *E*. *coli*. Proteolytic removal of the SUMO moiety from the fusion proteins resulted in the assembly of VLP with size and shape resembling the authentic FMDV. Immunization of guinea pigs, swine and cattle with FMD VLP by intramuscular inoculation stimulated the FMDV-specific antibody response, neutralizing antibody response, T-cell proliferation response and secretion of cytokine IFN-γ. In addition, immunization with one dose of the VLP resulted in complete protection of these animals from homologous FMDV challenge. The 50% protection dose (PD_50_) of FMD VLP in cattle is up to 6.34. These results suggest that FMD VLP expressed in *E*. *coli* are an effective vaccine in guinea pigs, swine and cattle and support further development of these VLP as a vaccine candidate for protection against FMDV.

## Introduction

Foot-and-mouth disease (FMD) is an acute, highly contagious viral disease, which may cause severe economic losses in susceptible cloven-hoofed animals [1,2]. In 1997 an FMD outbreak was reported in Taiwan which had been free of the disease for 68 years. This devastating outbreak resulted in the slaughter of more than 4 million pigs, almost 38% of the entire pig population, at a cost of approximately U.S. $6 billion [3]. In 2001 a large FMD outbreak started in the United Kingdom [4] and spread to France, Ireland and The Netherlands. The losses to agriculture in the United Kingdom alone were about £3.1 billion [5]. These outbreaks have significantly increased public awareness of this highly infectious disease and highlight the importance of disease control, including regular vaccination and slaughter of infected and contact animals, in endemic areas [6,7]. Although the conventional inactivated FMDV vaccine has been extremely successful in reducing the number of disease outbreaks in many parts of the world where the disease is enzootic, there are a number of concerns and limitations with its use in emergency control programs. Among these concerns and limitations, safety issues such as the possibility of virus escape during vaccine production and through insufficient chemical inactivation of virus highlight the need for the development of new generation vaccines.

Recently, virus-like particles (VLP) are increasingly recognized as safe, effective vaccine candidates for viral diseases [8]. VLP are virus-sized particles with the supra-molecular structures that have the form of rods or icosahedrons [9]. They are composed of multiple copies of one or more recombinant expressed viral structural proteins which spontaneously assemble into particles without incorporation of the viral genome. They display antigens in an ordered and repetitive way, thus inducing rapid, robust humoral immune responses as well as efficient T-cell responses [10]. Because VLP combine many of the advantages of whole virus vaccines and recombinant subunit vaccines into one system, two VLP vaccines, including the papillomavirus vaccine and the hepatitis B virus vaccine, are already licensed for use in humans, and a number of other VLP vaccines are being tested [8]. Many attempts have also been made to produce FMD VLP in a variety of hosts. The VLP-based FMD vaccines produced in baculovirus/insect cells, baculovirus/silkworm larvae, and by a replication-deficient human adenovirus vector-based system are two of the successful recombinant vaccine candidates in protecting the target species (cattle or swine) against FMDV challenge [11-20]. The FMD VLP vaccine candidate produced by the deficient adenovirus is currently being manufactured in experimental batches and tested in cattle in the US mainland (for the first time in US history) as part of the veterinary licensing process [20]. Lee et al. reported the VLP produced in bacteria [21]. However, the immunogenicity of this VLP has not been evaluated. Compared with eukaryotic expression systems, such as insect and mammalian cells, expression of heterologous genes in bacteria is by far the simplest and most inexpensive means available for research or commercial purposes. However, only > 20% of heterologous genes expressed in *E*. *coli* render soluble or correctly folded proteins [22]. In order to improve the expression efficiency of heterologous proteins in *E*. *coli*, many modifications have been made, including the development of strong promoters [23], coexpression with chaperones [24] and use of protein fusions. Among them, expression of the recombinant proteins such as fusion proteins, especially for difficult-to-express proteins, is one of the most effective ways in improving the solubility of recombinant proteins. Several gene-fusion systems, such as NusA, maltose binding protein (MBP), glutathione-S-transferase (GST), ubiquitin (UB), and thioredoxin (Trx), have been developed [25,26]. These fusion proteins are frequently employed to enhance protein expression and to facilitate purification [27-29]. Recently, small ubiquitin-like modifier (SUMO) protein, a ubiquitin-related protein, has emerged as an effective biotechnological tool, since SUMO usually promotes correct folding and structural stability of the fusion proteins, leading to enhanced functional production of the partner proteins compared to its untagged version [30].

The FMDV genome is a positive-sense, single-stranded RNA with one large open reading frame. Its translation yields a polyprotein that is subsequently processed by virus-encoded proteases to produce structural and non-structural proteins necessary for virus assembly and replication. One of the initial polyprotein cleavage events, mediated by the 2A protein, is co-translational cleavage at the N terminus of the 2B protein. The P1-2A precursor is processed by a viral 3C protease to produce the structural proteins VP0, VP1 and VP3. These proteins can then self-assemble to form icosahedral empty capsid particles [31-33], which consist of 60 copies of each protein. Many attempts have been made to produce FMDV capsid protein(s) and subsequently assemble into VLP in eukaryotic expression systems by combining the P1-2A and 3C, in order to assemble virus capsids correctly [12,34]. However, this strategy has not been achieved in prokaryotic expression systems, especially in *E. coli*. To overcome this technical bottleneck, we recently developed an improved SUMO fusion protein system in *E. coli* according to previous research [21] and produced several water-soluble virus capsid proteins. Fusion proteins of three FMDV capsid proteins, VP0, VP1 and VP3, were efficiently expressed by the SUMO expression system in *E. coli*. After removal of the SUMO moiety from the fusion proteins, the three capsid proteins could be assembled into VLP with size and shape resembling the authentic FMDV. These VLP were prepared and used as an immunogen in guinea pigs, swine and cattle. The data show that FMDV-specific antibodies, neutralizing antibodies, T-cell proliferation and secretion of IFN-γ were efficiently induced in the immunized animals. Furthermore, the immunized animals were totally protected against challenge with FMDV after inoculation with one dose of the VLP. These results encourage further work towards the development of a VLP vaccine against FMDV.

## Materials and methods

### Cells and virus

BHK-21 cells were cultured at 37°C in a 5% CO_2_ atmosphere in Dulbecco’s modified Eagle’s medium (DMEM; Gibco, Invitrogen corporation, Grand Island, NY, USA) supplemented with 10% fetal bovine serum (FBS; HyClone Laboratories Inc., Victoria, Australia), 100 U/mL penicillin and 100 mg/mL streptomycin. FMDV strain Asia1/Jiangsu/China/2005 (GenBank accession number: EF149009) was propagated in BHK21 cells.

### Plasmid constructions

A SUMO fusion protein expression vector was constructed as described previously with modification [35]. This vector is designated as pSMK which carries a kanamycin resistant gene (KanR). A pSMA vector that carries an ampicillin resistant gene (AmpR) and a pSMC vector that carries a chloramphenicol resistant gene (ChlR) were constructed by replacing KanR of the pSMK vector with AmpR and ChlR, respectively. Three FMDV capsid proteins VP0, VP1 and VP3 were amplified by polymerase chain reaction (PCR), subcloned into pSMK, pSMA and pSMC respectively. The recombinant plasmids were designated as pSMKVP0, pSMAVP1 and pSMCVP3 respectively.

### Protein production

To simultaneously express the three SUMO fusion proteins in the same *E. coli* cells, pSMKVP0, pSMAVP1 and pSMCVP3 were transformed into *E. coli* BL21(DE3) (Stratagen, La Jolla, CA, USA) simultaneously and selected by Amp, Kan and Chl resistance. Expression, purification and proteolytic cleavage of His6-Smt3 fusion proteins were carried out as described before [35]. The expressed proteins were analyzed by 10% sodium dodecyl sulphate polyacrylamide gel electrophoresis (SDS-PAGE) under denaturing conditions and the specificity of the proteins was confirmed by immunoblot assay [12].

### VLP quantification

A sucrose gradient ultracentrifugation method was used for analytical purpose as previously described [31]. Briefly, the proteolytically cleaved protein or inactivated virus was layered on the top of a 10%–30% (w/v) sucrose gradient in NET buffer (0.1 M NaCl, 0.001 M EDTA, 0.05 M Tris–HCl, pH 7.5). The samples were then centrifuged at 35 000 rpm for 3 h using an Optima L-100 XP ultracentrifuge (Beckman Coulter, Fullerton, CA, USA). After centrifugation, the gradients were fractionated and the optimal density (OD) at 280 nm or 260 nm of each fraction was measured using UA-Visible Spectrophotometer BioMate 3S (ThermoFisher Scientific Inc. Madison, WI, USA) and the quantity of intact capsids was determined. The fraction was used for electron microscopy.

### Electron microscopy observation of VLP

Samples for transmission electron microscopy (TEM) measurements were prepared by dipping a drop of the purified protein complex onto Formvar coated copper grids (300 mesh, Pelco, CA, USA) at room temperature. The grid was then removed, and excess liquid was drained off by blotting the edge of the grid with a piece of clean filter paper. Next, the grid was floated on a drop of 2% phosphotungstic acid (pH 6.5) for 1 min and air-dried for a few minutes after the excess phosphotungstic acid was removed as before. TEM images were recorded on a JEOL 2010 transmission electron microscope operated at an acceleration voltage of 100 kV.

### Animal immunization and challenge protocols

Fifteen guinea pigs weighing 400–500 g were obtained from the laboratory animal center of Lanzhou veterinary research institute, China. All the animals were fed in an isolated hutch. The guinea pigs were randomly divided into three groups of 5 animals each: group A, inactivated FMDV serotype Asia1; Group B, FMD VLP; Group C, phosphate buffered saline (PBS, pH 7.4). The guinea pigs were injected with 0.2 mL inactivated FMDV or 50 μg FMD VLP together with unassembled proteins emulsified by freund complete adjuvant or 0.2 mL PBS in the tibialis cranialis muscle of both rear legs. The serum samples were taken from the heart at 28 days post-immunization (dpi) after guinea pigs were anesthetized with pentobarbital sodium. All guinea pigs were subcutaneously and intradermally challenged with 0.2 mL 100 times 50% infective dose (100 ID_50_) per guinea pig of homologous live virus on left back sole at 28 dpi. All guinea pigs were kept in an isolated hutch and examined for 7 days. The lesion appearing only on the left back sole was referred to as an indicator of partial protection, on both back soles as an indicator of no protection and no lesion on the back was considered as an indicator of total protection.

Thirteen 2-month-old pigs, sero-negative for FMDV, were purchased from a conventional breeding/finishing farm. All animals were housed in an animal biosafety level 3 (ABSL3) facility and divided into three groups. There are five pigs in Group A and Group B, three pigs in Group C. Group A: Inactivated FMDV serotype Asia1 (2 mL); Group B: FMD VLP (50 μg); Group C: PBS (2 mL). FMDV inactivated vaccine or FMD VLP were emulsified with the adjuvant Montanide ISA 206 (Seppic, Paris, France) with a ratio of 1:1. All pigs were intramuscularly inoculated at the ear-root-neck area and bled at 10, 18 and 28 dpi. Four weeks after vaccination, all pigs were challenged by direct inoculation at the ear-root-neck area with the virulent homologous virus strain Asia1/Jiangsu/China/2005 at 1000 ID_50_ per pig. All pigs were housed in an isolated facility and examined for 10 days after challenge. The animals were examined daily for clinical signs of FMD, including the appearance of vesicles on the mouth and feet. Any lesion on the snot and feet of pigs was referred to as an indicator of no protection.

Seventeen one-year old cattle, sero-negative of FMDV, were divided into four groups. Group A: one dose of FMD VLP; Group B: 1/3 dose of FMD VLP; Group C: 1/9 dose of FMD VLP; Group D: Healthy control. Except for Group D which included 2 cattle, other groups included 5 animals each. All animals were housed in a BSL3 facility and were immunized by intramuscular inoculation of FMD VLP emulsified with the adjuvant Montanide ISA 206 with the ratio of 1:1. One dose contains 50 μg FMD VLP. Fifty percent protection dose (PD_50_) test was performed as described by the OIE to test the potency of FMD VLP as a vaccine candidate. Vaccinated and control cattle were challenged by tongue intradermal inoculation with a homologous virus strain Asia1/Jiangsu/China/2005 at 10 000 ID_50_ per head. The animals were periodically inspected for possible occurrence of lesions in the mouth, tongue, lips, buccal mucosa, teats, and feet. Any lesion at a site in the mouth other than the inoculation site within 10 days post-challenge (dpc) was considered as a rupture of immunity and considered as no protection. Blood and serum samples were collected by standard protocols at 9, 16, 21 dpi and 2, 5, 8, 10 dpc. The bovine PD_50_ content of the vaccine was calculated based on the Reed-Muench method from each animal protected in each group.

### Ethics statement

All animals received humane care in compliance with good animal practice according to the Animal Ethics Procedures and Guidelines of the People's Republic of China. The specific experiments were approved by Animal Ethics Committee of Lanzhou Veterinary Research Institute, Chinese Academy of Agricultural Sciences (permit number LVRIAEC2011-018).

### Western blot analysis of animal serum

BHK-21 cells in serum-free DMEM were infected with FMDV type Asia1/Jiangsu/China/2005 at a multiplicity of infection of 10 and incubated for several hours at 37°C with 5% CO_2_ until a cytopathic effect (CPE) was observed by light microscopy. Cells uninfected and infected with FMDV Asia1/Jiangsu/China/2005 were harvested and frozen-thaw for three times. 5 × SDS loading buffer was added in cell lysates and boiled for 10 min. The specificity of animal serum against whole virus or VLP was confirmed by immunoblot assay using sera obtained from animals unvaccinated or vaccinated with inactivated FMD vaccine and VLP vaccine, respectively.

### Determination of antibody titers by ELISA

The antibody titers in immunized guinea pigs were determined by indirect-ELISA as described elsewhere [36,37]. Briefly, 96-well plates were coated with inactivated FMDV Asia1/Jiangsu/China/2005 (100 mL/well) in 0.05 M bicarbonate buffer (pH 9.6) at 4°C overnight. After binding of the target antigen, wells were blocked in 100 μL PBST containing 1% BSA at 37°C for 1 h, washed and drained. One-hundred fold dilutions of sample sera were added to the microtiter wells in 50 μL of PBST-BSA and incubated for 1 h at 37°C. After the sample sera were removed and washed. Horseradish peroxidase (HRP)-conjugated anti-guinea pig antibody (1:2000) (Sigma, St. Louis, MO, USA) was added and incubated for 1 h at 37°C. Then the enzyme substrate o-phenylenediamine (OPD, Sigma, St. Louis, MO, USA) in 50 μL sodium citrate was added to each well and incubated for 15 min at room temperature. The reaction was stopped with 50 μL 2 M H_2_SO_4_, and the plate was read at 492 nm on a spectrophotometer (BioRad, Hercules, CA, USA). Antibody reactivity was reported as OD values.

The FMDV-specific antibody titers in immunized pigs and cattle were detected by a liquid-phase-block ELISA (LPB-ELISA) kit as described by Shao et al. [38]. Briefly, 50 μL of 2-fold serial dilution of each test serum in duplicate were prepared in U-bottom multiwell plates (Corning, NY, USA). To each well, 50 μL of a constant dose of viral antigen that was used to raise the rabbit antiserum for coating the plates were added and left at 4°C overnight. Subsequently, 50 μL of the serum-antigen mixture were transferred to an ELISA plate pre-coated with rabbit anti-FMDV serum and incubated at 37°C for 1 h at a dilution of 1:1000. After thorough washing with PBST, 50 μL of guinea-pig antiserum was added to each well and the plates were incubated at 37°C for 1 h. The plates were washed five times with PBST, followed by the addition of 50 μL of rabbit anti-guinea pig IgG-HRP at a dilution of 1:2000 (Sigma) and incubated at 37°C for 1 h. Then, the enzyme substrate o-phenylenediamine (Sigma) was added to each well for 10 to 15 min of incubation after being washed and drained. The reaction was terminated with 2 M H_2_SO_4_, and the plate was read at 492 nm on a spectrophotometer (BioRad, Hercules). Antibody titer was reported as log10 of the reciprocal of the highest dilution.

### Serum neutralization assay

Serum samples collected from animals at different time points were heat-inactivated (30 min, 56°C) and used in a microtiter neutralization assay on BHK-21 cells. Serial dilutions of serum were incubated with 100 TCID_50_ of FMDV strain Asia1/Jiangsu/China/2005 at 37°C and 5% CO_2_ for 1 h, followed by infection of monolayers of BHK-21 cells in 96-well plates for 72 h. Thereafter, the cells were examined for FMDV-specific cytopathic effect and neutralization titers were calculated as log10 of the reciprocal of the highest dilution resulting in 50% neutralization [36].

### Lymphocyte proliferation assay

The T-lymphocyte proliferation assay was performed with the Cell Titer 96AQueous Non-Radioactive Cell Proliferation Assay (Promega, Madison, WI, USA). Five guinea pigs from each group were sacrificed and single lymphocyte suspensions were prepared from spleens at 28 dpi as described previously [36,37]. Peripheral blood mononuclear cells (PBMC) were isolated from pigs at 28 dpi or cattle at 21 dpi by centrifugation in Ficoll-Paque Plus (density 1.077; Amersham Biosciences, Corston, UK) at room temperature for 30 min. Mononuclear cells were collected from the buffy coat and centrifuged, and residual red blood cells were lysed by incubation in water for 1 min followed by the addition of Eagle’s solution. After two washes in PBS, the cells were resuspended in RPMI 1640 supplemented with 25 mM HEPES, 2 mM glutamine, 10% FBS, penicillin/streptomycin. Lymphocyte suspensions of spleen or PBMC were added to 96-well flat-bottomed plates at a concentration of 100 μL per well (2 × 10^5^ cells per well). Subsequently, 100 μL per well of medium with or without inactivated FMDV Asia1/Jiangsu/China/2005 (10 μg/mL) was added and mixed. Each sample was tested in triplicate. Phytohaemagglutinin (PHA) (Sigma) at the final concentration of 10 μg/mL was used as a positive control. The plates were incubated at 37°C for 60 h followed by incubation with a tetrazolium compound [3-(4,5-dimethyl-2-yl) -5- (3-carboxymethoxyphenyl)-2-(4-sulfophenyl)-2H-tetrazolium, inner salt; MTS] and then incubated at 37°C under 5% CO_2_ for 4 h. The absorbance at 490 nm was measured with a spectrophotometer (BioRad, Hercules, CA, USA). Data are expressed as stimulation index (SI), calculated as the mean reading of triplicate wells of antigen-stimulated cells divided by the mean reading of triplicate wells from unstimulated (negative control) wells.

### Analysis of cytokine IFN-γ

Spleen lymphocyte or PBMC supernatants were cultured with 10 μg/mL of inactivated FMDV Asia1/Jiangsu/China/2005 for 72 h and analyzed for cytokine IFN-γ expression using commercially available guinea pig, porcine, bovine IFN-γ ELISA kits (Uscn Life Science, Wuhan, China) by following the instructions supplied. The concentration of IFN-γ in the samples is then determined by comparing the O.D. of the samples to the standard curve.

### Virus infectivity assay

The presence of virus in serum was determined by a standard plaque assay on BHK-21 cells. Briefly, confluent monolayers of cells on six-well plates were adsorbed with serial dilutions of serum samples. Following adsorption at 37°C for 1 h, the samples were removed and cells were washed twice with PBS (pH, 7.4). Then, 2 mL of DMEM containing 1% agarose gel were added and incubated at 37°C in a humidified atmosphere containing 5% CO_2_ for 24 h. Cells treated with samples were stained with a neutral red solution to visualize the plaques. Virus titers were expressed as log10 PFU per mL.

### Detection of FMDV RNA by real time RT-PCR

Frozen serum samples from animals were thawed and processed for RNA extraction and measurement of specific FMDV RNA by real-time reverse transcription-PCR (RT-PCR) as previously described. Real time RT-PCR was performed using a Mx3005P sequence detection system (Agilent, Santa Clara, CA, USA). Samples were considered negative when FMDV RNA molecules/mL were less than 10^3^[39,40].

### Statistical analysis

Data are presented as mean ± SD. The statistical analysis was first performed to verify the homogeneity of variance by using the Levene test. Then, the analysis of variance between groups using One-way ANOVA was applied. Finally, a comparison of mean pair wise differences between groups using Least Significance Difference (LSD) was performed. Significance of all statistical tests was set at 0.05 (*p* < 0.05).

## Results

### Construction and expression of soluble His6-SM FMDV capsid proteins

**Figure 1:**
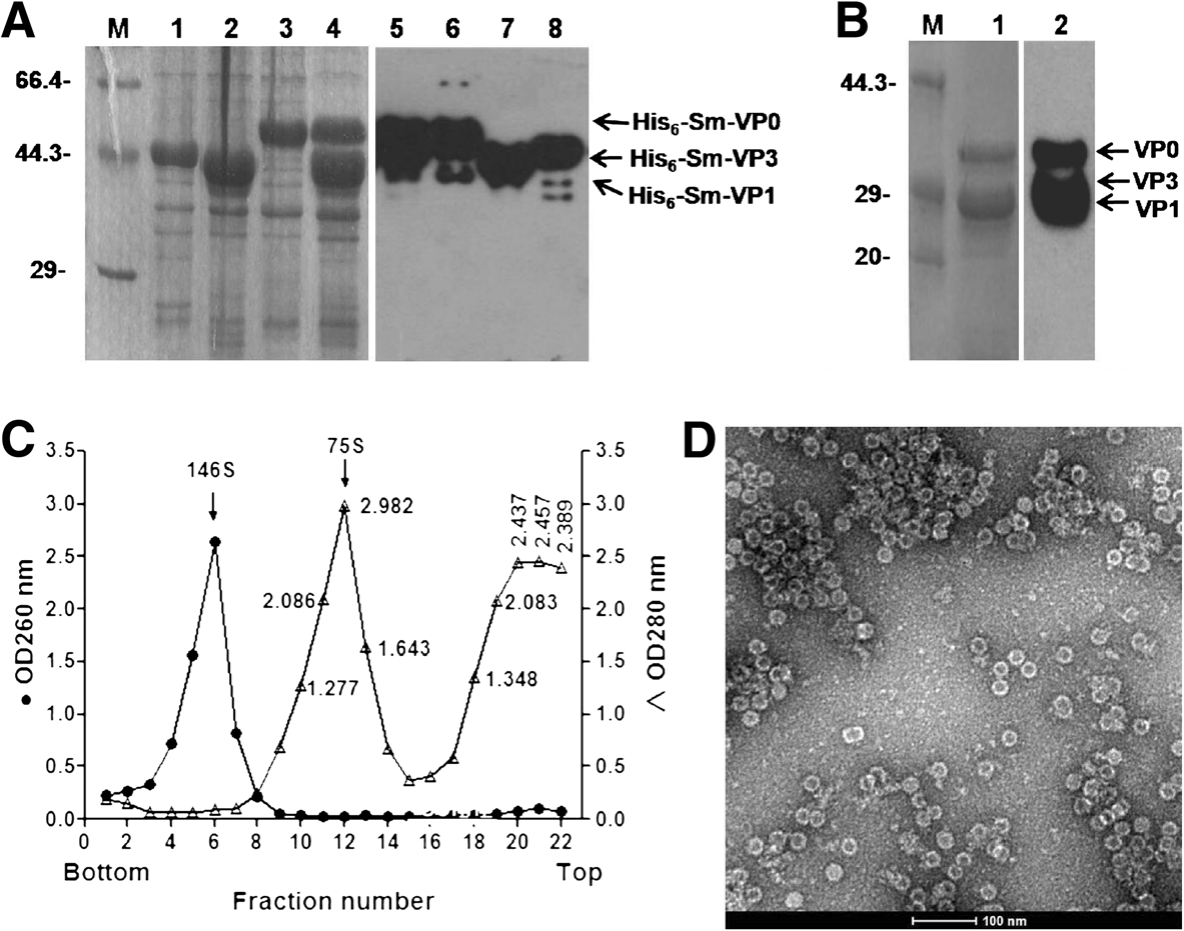
**Expression and assembly of purified FMDV capsid proteins. (A)** SDS-PAGE (left panel, lanes M, 1–4) and Western blot (right panel, lanes 5–8) analysis of the purified capsid protein complexes without Ulp1 protease treatment, including the SUMO-tagged FMDV VP3 protein expressed individually (lanes 1 and 8), the SUMO-tagged FMDV VP1 protein expressed individually (lanes 2 and 7), the SUMO-tagged FMDV VP0 protein expressed individually (lanes 3 and 6) and the three SUMO-tagged protein complex simultaneously expressed (lanes 4 and 5). **(B)** SDS-PAGE (lane 1) and Western blot (lane 2) analysis of the purified three SUMO-tagged protein complex after protease digestion and the second affinity chromatography. **(C)** Purification and analysis of FMD VLP by sucrose gradient ultracentrifugation. The purified three capsid proteins were loaded onto a 10%–30% (w/v) sucrose gradient. After centrifugation, 20 fractions were collected and measured at 280 nm using UA-Visible Spectrophotometer BioMate 3S (ThermoFisher Scientific Inc. USA) and the quantity of capsid proteins in each fraction was determined and plotted. **(D)** Negative staining electron microscopy of FMD VLP containing purified VP0, VP3 and VP1. Scale bar, 100 nm.

Three components of the structural proteins of FMDV, VP0, VP1 and VP3, cleaved from the P1 protein precursor, can remain associated in a protein complex and act as a monomer for the self-assembly of five monomers into the pentameric capsid subunit. Further assembly of 12 pentamers and a molecule of genomic RNA generates a provirion [41,42], which subsequently undergoes maturation cleavage to convert VP0 into VP2 and VP4 to produce the mature infectious virion [43-45]. However, FMDV has the ability to form empty capsids sharing the same antigenicity as virions, but does not contain RNA and cleaved VP0 [46-48]. To express the three structural proteins and to further facilitate the assembly of FMD VLP, recombinant plasmids encoding VP0 (pSMKVP0), VP1 (pSMAVP1) and VP3 (pSMCVP3) were constructed. These three vectors expressing SUMO fusion proteins were simultaneously transformed and induced to express in the same *E. coli* host cells. As shown in Figure 1A, the three SUMO fusion proteins were all strongly induced by IPTG and were water-soluble. The expression efficiency was further improved by incubation at 16°C overnight, compared with the conditions at 37°C for 3–4 h (data not shown). The yield of the purified protein produced by the recombinant bacterial clone varied between 15–20 mg/L culture, which is significantly higher than the purified protein produced by the scheme described previously [21]. The molecular masses of His6-Sm-VP0, His6-Sm-VP1 and His6-Sm-VP3 fusion protein are approximately 45, 35 and 36 kDa, respectively (Figure 1A). So, the latter two protein bands were not well separated (Figure 1A, lane 4 and 5).

Extracts containing the coexpressed SUMO fusion proteins were purified on a Ni_2_^+^-resin and then treated with SUMO protease to remove the His6-Sm moieties. The cleavage products contained three water soluble polypeptides with molecular weights about 33 kDa (VP0), 24 kDa (VP3) and 23 kDa (VP1) and His6-Sm tag, respectively, upon examination by SDS-PAGE (data not shown). After purification of the cleavage products by Ni_2_^+^-resin again, the three fusion proteins were left in solution (Figure 1A).

### VLP assembly and quantification

To demonstrate if the expressed FMDV capsid proteins could assemble into empty particles, the proteolytically cleaved proteins were analyzed by sedimentation on a 10%–30% sucrose gradient. Fractions were collected and measured with a spectrophotometer. The results (Figure 1C) show the presence of a peak corresponding to 75S empty capsid particles from FMDV [12,31]. The peak fractions were pooled and the percentage of VLP was calculated from the peak area. This portion was approximately 43% of the total expressed proteins (Figure 1C), while the other 57% of proteins failed to assemble into stable empty capsids, as they remained near the top of the gradient (Figure 1C).

Transmission electron microscopy was then used to examine the extent of VLP assembly by these ternary protein complexes. As shown in Figure 1D, the VP0-VP1-VP3 complexes formed uniform round VLP aggregates with a diameter about 25 nm, which is similar to the average diameter of 25 nm of the authentic FMDV particles.

### Immunization with FMD VLP induces specific protective responses in guinea pigs

Guinea pigs are one of the ideal experimental animals for FMDV study and widely used for initial characterization of the antigenic properties of FMD vaccine candidates. To determine if FMD VLP could stimulate anti-FMDV immune responses in guinea pigs, groups of five guinea pigs were immunized with FMD VLP intramuscularly and two other groups of five animals were immunized with inactivated FMDV as a positive control and PBS as a negative control, respectively. The results show that FMDV-specific antibodies, neutralizing antibodies, specific T-cell response and IFN-γ responses were efficiently induced by immunization with FMD VLP. Significant differences were observed between guinea pigs inoculated with PBS and with either FMD VLP or FMD inactivated vaccine, however, no significant difference was found between guinea pigs inoculated with the FMD VLP and FMD inactivated vaccine.

After challenge with 100 ID_50_ FMDV Asia1/China/Jiangsu/2005 per guinea pig, all of the control animals inoculated with PBS showed typical symptoms on both back feet at 2 dpc, as expected. In contrast, none of the guinea pigs vaccinated with the inactivated FMD vaccine and the FMD VLP developed any symptom associated with FMDV replication.

### Immunization with FMD VLP induces specific protective responses in pigs

**Figure 2:**
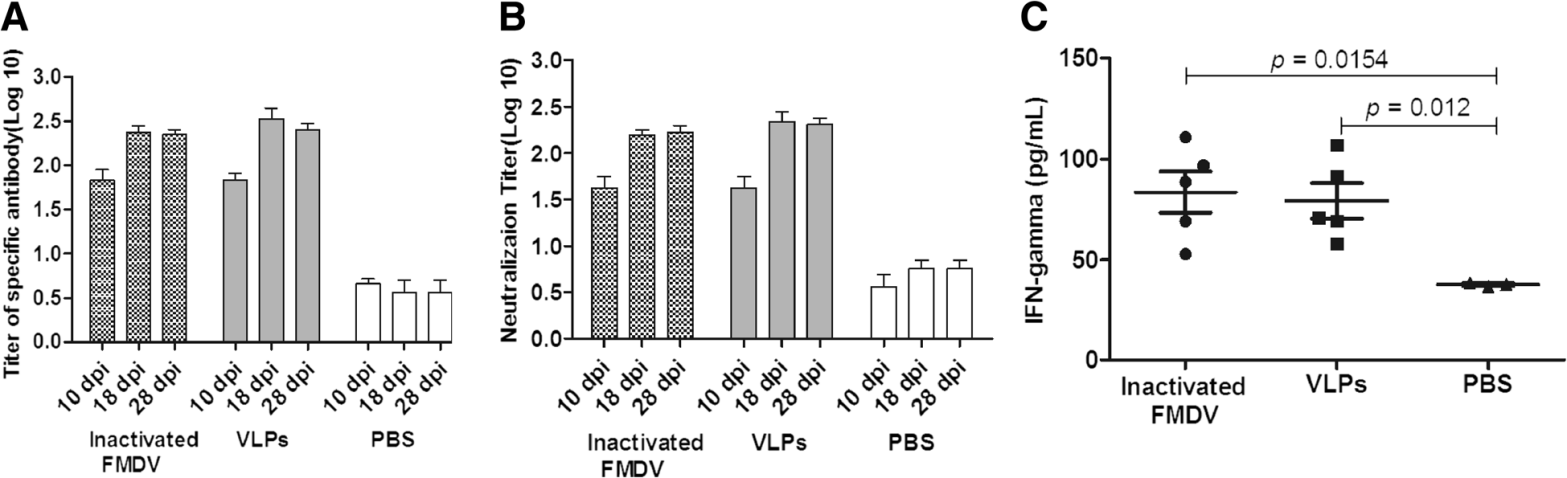
**FMDV-specific immune responses in pigs.** Groups of pigs (*n* = 5) immunized with inactivated FMDV or FMD VLP, including three PBS-inoculated pigs as a control, by intramuscular injection. Blood samples were collected at 10 dpi, 18 dpi and 28 dpi after vaccination for virus-specific antibody response **(A)** and neutralizing antibody response **(B)**. The titers of virus-specific antibody and neutralizing antibody were expressed by the average titers of pigs in one whole group at different time points. PMBC were isolated from all pigs at 28 dpi. IFN-γ secretion **(C)** by PMBC was measured and is shown as pg/mL. Statistical significance among groups was compared.

The efficiency of FMD VLP as a potent FMD vaccine candidate in guinea pigs prompted us to investigate the effectiveness of FMD VLP as a vaccine in pigs, one of the natural hosts of FMDV [49]. The FMDV-specific antibody responses were quantified by LPB-ELISA in pigs inoculated with FMD VLP. Compared to the control group inoculated with PBS, the presence of specific antibodies was detected at 10 dpi in all inoculated animals, and the level of specific antibodies increased and remained at a high level until 28 dpi (Figure 2A). FMDV-specific neutralizing antibodies were found in sera from pigs inoculated with the inactivated FMDV vaccine and the FMD VLP at 10 dpi (Figure 2A). All animals in the two groups showed high neutralization titers after the vaccine administration. Subsequently, the titers were further increased and remained at a high level until challenge (28 dpi), like the specific FMDV antibodies (Figure 2B). The average levels of the specific and the neutralizing antibodies in pigs inoculated with FMD VLP are similar to those in the group inoculated with the inactivated FMDV (Figure 2A and B), suggesting that FMD VLP are competent to induce specific humoral immune response as the whole FMDV particles in pigs.

FMDV specific T-cell responses were detected in all animals vaccinated with both the FMD VLP and the inactivated FMDV at 28 dpi. Although individual pigs showed high levels of T-cell response, the average proliferative responses of all vaccinated pigs did not show a highly significant increase in the stimulation indexes, compared with the animals inoculated with PBS, which was different from the T-cell response in guinea pigs.

The induction of IFN-γ in PBMC samples from vaccinated pigs at day 28 post-vaccination was also determined. As with the lymphoproliferative responses, secretion of IFN-γ did not show significant increase in pigs inoculated with the FMD VLP and the inactivated FMDV, compared with the PBS control group (Figure 2C). However, the levels of IFN-γ induction were consistent with the lymphocyte proliferative responses in individual pigs (data not shown).

The immunized pigs were challenged with 1000 ID_50_ FMDV Asia1/China/Jiangsu/2005 per pig at 28 dpi. Three animals in the PBS control group developed clinical signs of disease on all feet, which remained for several days. In contrast, none of the animals inoculated with either the FMD VLP or the FMDV inactivated vaccine showed any clinical signs in the mouth and feet. This confirms that FMD VLP can provide efficient protection in pigs against FMDV as the conventional inactivated FMD vaccine.

### Immunization with FMD VLP induces specific protective responses in cattle

**Figure 3:**
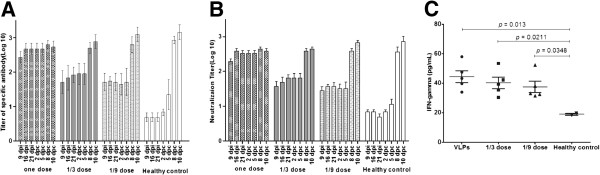
**FMDV-specific immune responses in cattle.** Groups of cattle (*n* = 5) immunized with one dose, 1/3 dose, 1/9 dose of FMD VLP, including two healthy cattle as the control, by intramuscular injection. Blood samples were collected at 9 dpi, 16 dpi, 21 dpi and 2 dpc, 5 dpc, 10 dpc for virus-specific antibody response **(A)** and neutralizing antibody response **(B)**. The titers of virus-specific antibody and neutralizing antibody were expressed by the average titer of cattle in one whole group at different time points. PMBC were isolated from all cattle at 21 dpi. IFN-γ secretion **(C)** by PMBC was measured and shown as pg/mL. Statistical significance among groups was compared.

The potency of the immunity induced by the FMD VLP in cattle was evaluated as recommended in the OIE manual [50]. Three groups of cattle were inoculated with one dose, 1/3 dose and 1/9 dose of FMD VLP, and LPBE-antibody titers were determined at 9, 16, 21 dpi and 2, 5, 8, 10 dpc, respectively. All animals vaccinated with FMD VLP developed a detectable, dose-dependent FMDV-antibody response at 9 dpi, which reached to a higher level at 16 dpi (Figure 3A). By 21 dpi, the antibody levels were either maintained at the same level in the groups inoculated with one and 1/9 dose, or slightly increased in the group inoculated with a 1/3 dose (Figure 3A). Throughout this period, the antibody levels in the healthy control group remained low (Figure 3A). Except in cattle inoculated with one full dose of FMD VLP (which shows a slight increase of the antibody titer after challenge), drastic induction of specific antibody responses was observed in all other three groups of cattle after challenge at 8 and 10 dpc (Figure 3A).

Similar patterns in serum neutralizing antibody responses were also observed. As shown in Figure 3B, the neutralizing antibody response in the group of cattle vaccinated with one dose of FMD VLP was significantly higher than that in cattle inoculated with 1/3 or 1/9 dose of FMD VLP, and a gradual increase of neutralizing antibody response over time was observed in most vaccinated cattle (Figure 3B). Following challenge, the neutralizing antibody titers in cattle inoculated with one dose of FMD VLP remained at the same high level, while a rapid serum-conversion was evident in the other three groups of animals vaccinated with 1/3 dose, 1/9 dose and healthy control at 8 dpc, respectively (Figure 3B). However, there was no statistically significant difference in titers for all the treatment groups by 10 dpc (Figure 3B). This result was in agreement with the LPBE-antibody titers in cattle.

Dose-dependent lymphoproliferation responses were detected in all groups vaccinated with FMD VLP; however, only animals in the group inoculated with one dose and 1/3 dose of FMD VLP displayed an increase in T-cell response, compared with the healthy control (data were not shown). Similarly, the production of IFN-γ in PMBC from vaccinated cattle at 21 dpi also displayed a significant dose-dependent induction pattern, compared with the healthy control (*p* < 0.05) (Figure 3C).

### Detailed evaluation of the protection potency of FMD VLP in cattle

**Figure 4:**
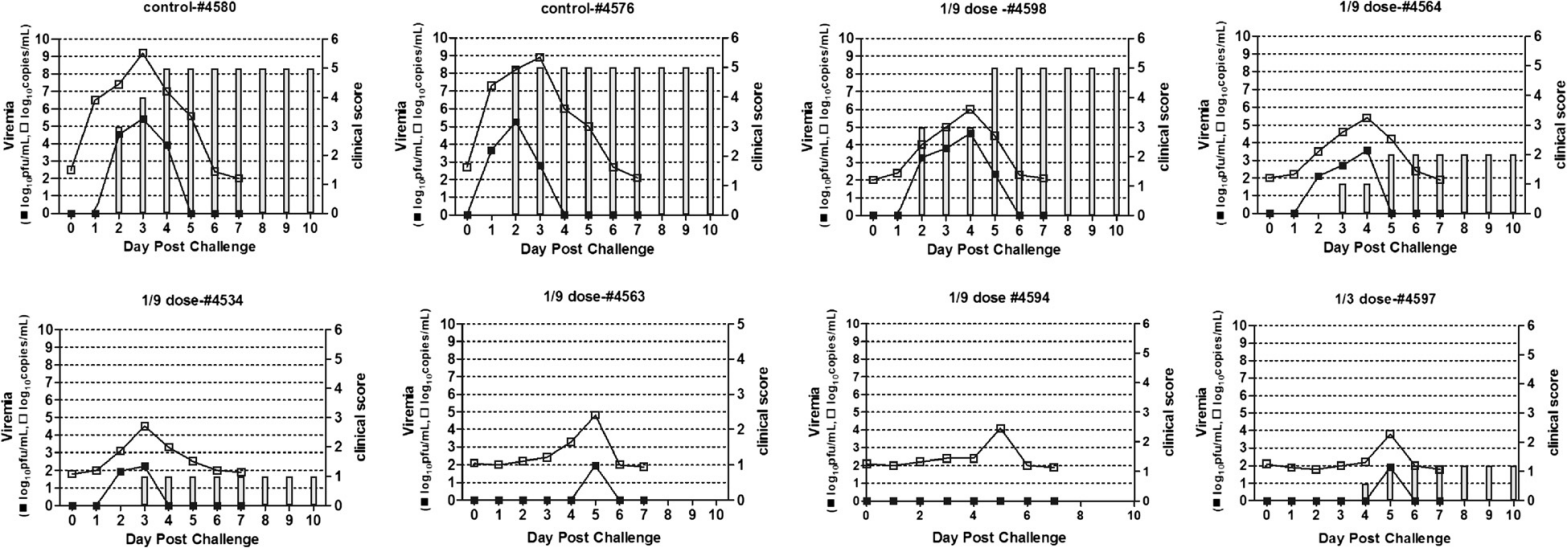
**The clinical outcome of cattle challenged with FMDV.** Cattle treated with one dose, 1/3 dose, 1/9 dose of FMD VLP and healthy cattle as control were challenged with 10 000 ID_50_ of FMDV Asia1/Jiangsu/China/2005 at 21 dpi. The animals were carefully examined in the muzzle, inside the mouth and feet every day for 10 days after challenge with the homogenous virus. A clinical score of 0 to 5 (right Y axis) was assigned to describe the severity of the disease, with a maximal score of 5: a score of 0 was assigned when one or more vesicular lesions were detected at the site of injection on the tongue; a score of 1 was assigned when one or more vesicular lesions were detected in the mouth other than the tongue; a score of 2 was assigned when one or more vesicular lesions were detected in one foot, a score of 3 was assigned if two feet had vesicular lesions, a score of 4 was assigned if three feet had vesicular lesions, a score of 5 was assigned if four feet had vesicular lesions. Animals showing viremia were selected and listed. The maximum clinical score (bars) is 5. Viremia (left Y axis) determined by infectivity and viral RNA level are expressed as log10 PFU/mL and log10 copies/mL respectively.

Animals in all groups described above were challenged by intradermal inoculation at two sites in the tongue with 10 000 ID_50_ FMDV Asia1/Jiangsu/China/2005 at 21 dpi. Both animals in the control group developed lesions at secondary sites of replication, including the feet, and more severe disease was seen by 2–10 dpc. All animals inoculated with one dose of FMD VLP developed a vesicle at the sites of injection only. The vesicle was self-limited and did not increase after 2 dpc. In the group inoculated with 1/3 dose of FMD VLP, one animal developed a delayed lesion, which was detectable by 4 dpc and was present on one foot. Three animals in the group inoculated with 1/9 dose of FMD VLP developed lesions beyond the injection sites (Figure 4).

Viremia was evaluated in all animals by determination of the infectivity (PFUs/mL) and the FMDV RNA levels in sera. The control animals (#4580, #4576) developed viremia at day 1 dpc which then lasted for 5 days (Figure 4). Cattle in the group inoculated with one dose of FMD VLP induced strong adaptive immune response, which conferred potent protection against challenge with FMDV serotype Asia1 and limited the replication of FMDV in the challenged animals. No viremia was detected in this group of animals, both by the plaque assay and by real time RT-PCT (Figure 4). Viremia was detected in one of the animals inoculated with 1/3 dose of FMD VLP, but not detected in other animals in the group (Figure 4). All the animals inoculated with 1/9 dose of FMD VLP developed viremia, as detected by a plaque assay and confirmed by real-time RT-PCR, although one animal in this group did not show lesions (Figure 4). The onset of viremia in all animals in this group was delayed and the viremia lasted for a shorter period of time. The copies of FMDV RNA molecules were generally 10^3^- to 10^5^-fold lower than in the control animals (Figure 4).

The PD_50_ test was then performed to assess the subunit vaccine potency following the bovine potency test protocol for traditional inactivated FMD vaccines described by the OIE. The result shows that the vaccine potency of the batch immunized with the FMD VLP reached 6.34 PD_50_ per dose, which was higher than that for routine prophylactic use and also meets the requirement for emergency (“ring”) vaccination as specified by OIE (Table 1).

**Table 1:** The neutralizing titer, T-lymphocyte proliferation and protection of cattle

**Group**	**No**	**Neutralizing titer**			**T-lymphocyte proliferation (SI)**	**Protection**	**Rate of protection**	**PD50**
		**9 dpi**	**16 dpi**	**21 dpi**	**21 dpi**	**10 dpc**		
**One dose**	#4536	2.408	2.709	2.107	1.989	yes	100(5/5)	6.34
	#4572	2.107	2.408	2.408	1.602	yes		
	#4574	2.408	2.709	2.107	1.373	yes		
	#4590	2.107	2.408	2.408	1.586	yes		
	#4592	2.408	2.709	2.408	1.625	yes		
**1/3 dose**	#4501	1.806	1.806	1.806	1.587	yes	80(4/5)	
	#4561	1.204	1.505	1.806	1.512	yes		
	#4575	1.806	2.107	2.107	1.721	yes		
	#4593	1.806	1.806	1.806	1.889	yes		
	#4597	1.204	1.204	1.505	1.353	no		
**1/9 dose**	#4534	1.505	1.505	1.806	1.351	no	40(2/5)	
	#4563	1.505	1.505	1.204	1.886	yes		
	#4564	1.204	1.505	1.505	1.375	no		
	#4594	1.806	1.806	1.806	1.558	yes		
	#4598	1.204	1.505	1.505	1.397	no		
**Healthy cattle**	#4576	0.778	0.903	0.778	1.052	no	0(0/5)	
	#4580	0.903	0.778	0.602	1.115	no		

### Efficient induction of antibody responses by FMD VLP against the three structural proteins

**Figure 5:**
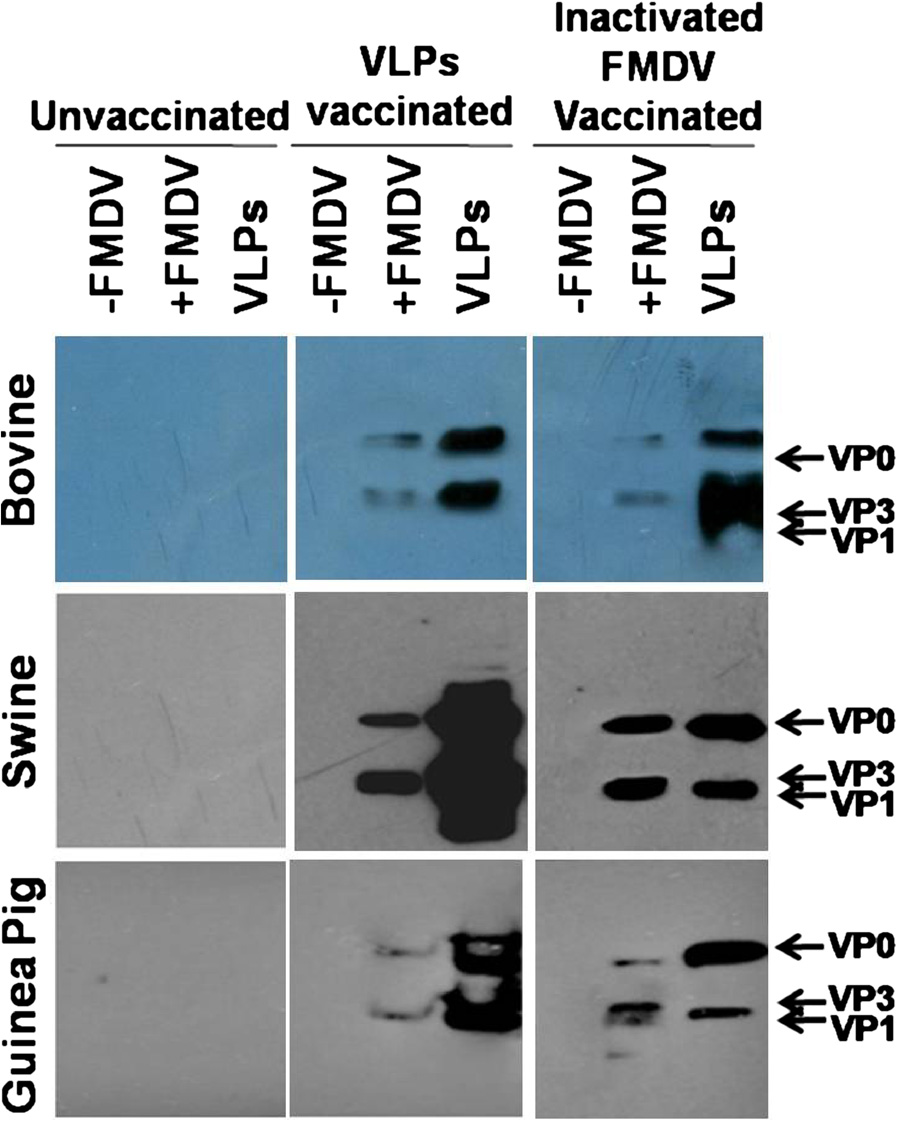
**Specificity of animal serum against whole FMDV and VLP.** The purified FMD VLP (about 0.1 μg) and total cell lysates prepared BHK-21 cells infected with mock or FMDV were separated by SDS-PAGE, and analyzed by Western blot with sera from cattle, pig and guinea pig unvaccinated or vaccinated with inactivated FMDV or FMD VLP being used as primary antibodies.

Sera from animals vaccinated with the FMD VLP and the inactivated FMDV vaccine were finally assessed by Western blot to investigate if all three structural proteins used to prepare the VLP are immunogenic. For this purpose, total cell lysates were prepared from FMDV-infected BHK-21 cells and analyzed by Western blot. The results show that sera collected from all three animal species vaccinated with FMD VLP could efficiently react with the three proteins from both virus-infected cells and FMD VLP preparation (Figure 5). Moreover, similar patterns and efficiencies of the reaction were observed from animal sera vaccinated with the inactivated FMDV (Figure 5). These results demonstrate that all three structural proteins are equally antigenic, although they are expressed and purified from a prokaryotic expression system, and further suggest that these proteins may be incorporated and assembled into VLP with an equal efficiency as well as in a correct conformation.

## Discussion

In this study, we attempt to produce the FMD VLP in *E. coli* and use this VLP preparation as a vaccine candidate for the first time to characterize the immunological properties in a laboratory animal model and in two natural FMDV host animal species, swine and cattle.

The three SUMO fusion proteins were shown to form stable VLP with size and shape resembling the native FMDV particles, upon simultaneous expression and removal of the SUMO moiety from the SUMO-fusion proteins. The SUMO-fusion system with modification provides increased levels of expression of FMDV capsid proteins in *E. coli* and allows rapid purification of target proteins, by exploiting the properties that SUMO fused at the N-terminus with other proteins can fold and protect the protein by its chaperoning properties. The results of this study demonstrate that SUMO-fusion dramatically enhances the expression of the FMDV capsid protein, which is the precondition for FMD VLP assembly. Although it is not fully understood why SUMO fusion can enhance the protein solubility, structural studies suggest that SUMO has an external hydrophilic surface and inner hydrophobic core, which may exert a detergent-like effect on otherwise insoluble proteins [30]. The next question would be whether the immunogenicity of these VLP is also similar to the FMDV native virions. Our results indicate that the FMD VLP could induce specific antibody responses against whole FMDV virions in guinea pigs, swine and cattle, as demonstrated by indirect ELISA, LPB-ELISA and Western blot analyses. This suggests that the three proteins expressed and purified from *E. coli* are properly folded in the VLP. In addition, the data available from mapping of the neutralizing antibody epitopes in serotype O, the most extensively studied FMDV [51,52] and in Asia 1 by Butchaiah and Morgan [53] demonstrate that the major immunologic epitopes of serotype Asia 1 are on the surface oriented interconnecting loops between structural elements. Our observation that potent neutralizing antibody responses were induced by FMD VLP in all three vaccinated animal species demonstrates that the three proteins were not only correctly assembled into VLP but the neutralizing epitopes were also presented correctly on the surface of virus capsids. Further confirmation of the neutralizing epitopes of FMDV Asia1 together with crystallographic studies to identify the structure of the FMDV capsids would provide more insight into these issues.

A neutralization test was carried out in this study after detection of specific antibodies against whole FMDV in sera from guinea pigs, pigs and cattle. Interestingly, the specific antibody titers in animals vaccinated with FMD VLP, as determined by indirect-ELISA and LPB-ELISA, showed a good correlation with the neutralization titers, which was consistent with the results reported previously [54,55]. Meanwhile, a good correlation between specific antibody titers and protection was also observed in this study.

Virus-induced neutralizing antibody response is crucial for the development of protection against FMD. A nice correlation between the neutralizing antibody titers and the levels of protection against live virus challenge has been found in many studies [56-58]. Similarly, neutralizing antibody responses in guinea pigs, pigs and cattle inoculated with either one or 1/3 dose of the FMD VLP were shown in this study to have a positive correlation with protection against FMDV challenge. However, at 21 days post vaccination (pre-challenge) in cattle, one of the animals (#4563) inoculated with 1/9 dose of FMD VLP was shown to have an antibody titer lower than the threshold titer, but showed resistance to challenge; whereas another one (#4534) with a higher titer did not. This may suggest that the correlation between virus neutralization titer and protection could mainly depend on the dose of FMD VLP used in addition to the possibility of an individual variation in resistance to infection by the virus. This correlation may be proportional within a certain range of dosage. If the dose is too low, no correlation between virus neutralization titer and protection would be observed, as in the case when cattle were vaccinated with 1/9 dose of VLP (#4563 and #4534). Other factors, including batch and strain of FMD vaccine used, the route of inoculation, the virus titer for challenge, and so on, could also affect the correlation between the protection rate and the specific antibody/neutralizing antibody titer.

Certain non-antibody-mediated immune mechanisms, such as T-cell activation and the cytokines they release, may also play a role [59]. The results of T lymphocyte proliferation response and cytokine release in animals vaccinated in this study suggest that 50 μg FMD VLP is enough to induce the T-lymphocyte proliferation response and induction of IFN-γ in the guinea pig, pig and cattle. When the dose is too low to induce sufficient protective neutralizing titers, the T-cell response would promote protection against the virus infection initially provided by neutralizing antibodies. Although the T-cell response and IFN-γ secretion in pigs and cattle did not increase as significantly as shown in guinea pigs, a correlation between T-cell response and IFN-γ secretion was observed in all vaccinated animals. It is assumed that optimal IFN-γ production requires the inclusion of an efficient T-cell epitope in a suitable configuration. IFN-γ is a major activator of macrophages, enhancing their antimicrobial activity and their capacity for processing and presenting antigens to T lymphocytes [60]. It has been reported that IFN-γ stimulates MHC expression in antigen-presenting cells and efficiently inhibits FMDV replication [61]. Therefore, better clinical protection conferred by the FMD VLP in this study, especially when no sufficient neutralizing antibody response was elicited, would be due to an efficient lymphoproliferative response and IFN-γ release. Further biochemical studies are required to understand the cooperative, redundant, or synergistic effects in the antiviral properties of T-cell response and this cytokine induced by FMD VLP.

The development of lesions and viremia in FMD VLP-inoculated cattle challenged with FMDV appears to follow a dose-dependent response pattern. One dose of the 50 μg FMD VLP efficiently prevented the development of disease, when the animals were exposed to the virus by direct inoculation in the epithelia of the tongue as recommended by the OIE in efficacy testing [50]. Most animals (4/5) treated with 1/3 dose of FMD VLP also did not develop lesions; only one animal in this group with a lower neutralizing antibody response showed mild lesions and short-lived viremia. Two animals vaccinated with 1/9 dose of FMD VLP had a slight delay in the appearance of viremia and development of the disease, compared to the control animals. In general, a good correlation between the development of viremia and the neutralizing antibody response was observed after FMDV challenge within the groups of cattle.

The PD_50_ value of the VLP vaccine is calculated from the number of cattle protected in each group. According to the OIE manual [50], when employed for routine prophylactic use, the FMD vaccine should contain at least 3 PD50 per dose for cattle. But for emergency vaccination, it should have 6 PD50 per dose for cattle. In our study, the FMD VLP contain 6.43 PD50 per dose for cattle, illustrating the potency of FMD VLP as a potential emergency vaccine. On the contrary, FMD VLP can prevent the development of local tongue lesions at the site of challenge, indicating high potency of FMD VLP as one of the vaccine formats. In addition, since potency resulting from cattle tests can be a good indicator for the vaccine applicability in other species, the potency of FMD VLP could be sufficient to endorse its use in pigs, which is the reason why the potency test in swine was not carried out.

Taken together, our present results show the potential of using FMD VLP produced in *E. coli* as a vaccine candidate. Vaccination with a single injection of 50 μg of proteins could elicit a high level of immune response, which is sufficient to protect guinea pigs, swine and cattle from virulent virus infection.

## Competing interests

The authors declare that they have no competing interests.

## Authors’ contributions

GHC and SSQ carried out most of the experiments and drafted the manuscript. LXT, LDX, YH and LJX critically revised the experiment design and manuscript. JY, YSL, YSH, SDH, WYQ and MJU helped with the experiment. LZX and GJH helped with the addition experiment and revision of this manuscript. All authors read and approved the final manuscript.
